# Histological artifacts associated with laser and electroscalpel gingivectomy: Case series

**DOI:** 10.7705/biomedica.6930

**Published:** 2023-09-30

**Authors:** Jennifer Orozco, David Rico, Lía Barrios, Vivi Hoyos, Pilar Blanco

**Affiliations:** 1 Programa de Odontología, Corporación Universitaria Rafael Núñez, Cartagena, Colombia Corporación Universitaria Rafael Núñez Programa de Odontología Corporación Universitaria Rafael Núñez Cartagena Colombia; 2 Grupo de Histopatología, Facultad de Medicina, Universidad de Cartagena, Cartagena, Colombia Universidad de Cartagena Grupo de Histopatología Facultad de Medicina Universidad de Cartagena Cartagena Colombia; 3 Medical Laser Latinoamérica, Santa Marta, Colombia Medical Laser Latinoamérica Santa Marta Colombia

**Keywords:** Gingivectomy, artifacts, histology, lasers, semiconductor, lasers, solid-state, Gingivectomía, artefactos, histología, láseres de semiconductores, láseres de estado sólido

## Abstract

**Introduction.:**

Over time, efforts have been invested in the design of new instruments that overcome the disadvantages of the gold standard instrument in surgery, the scalpel. As a result, electronic equipment has emerged such as the electric scalpel and laser devices. The available evidence on these instruments suggests that the tissue response is related to each instrument’s physical and biological cutting principles.

**Objective.:**

To compare the histological changes in gingiva samples associated with surgical cutting performed with a 940 nm diode laser, a 2780 nm erbium, chromium: yttriumscandium-gallium-garnet *(*Er,Cr:YSGG) laser, and an electric scalpel, by presenting a series of cases.

**Case presentation.:**

We present three cases of healthy patients undergoing cosmetic surgery. The clinical examination revealed exposure of a keratinized gingiva band greater than 4 mm, normal color and texture in gingival tissue, with a firm consistency and no bleeding on periodontal probing. Gingivectomy was indicated with the following protocols: Diode laser of 940 nm at 1 W, in continuous mode; Er,Cr:YSGG laser of 2780 nm at 2.5 W, 75 Hz, H mode, air 20, water 40, gold tip MT4); and electric scalpel in cutting mode at power level four. Gingival tissue samples were taken and stored in 10% formaldehyde for histological analysis.

**Conclusion.:**

All the evaluated cutting instruments generated histological changes produced by the thermal effect, the main ones being collagen coagulation and carbonization. The depth of thermal damage caused by the 2780 nm Er,Cr:YSGG laser was much lesser than that induced by the electric scalpel and the 940 nm diode laser.

## Resumen

**Introducción.:**

Históricamente se ha invertido esfuerzo en el diseño de nuevos instrumentos que superen las desventajas del estándar de referencia en cirugía, el bisturí. Como consecuencia de esto, han surgido equipos electrónicos como el electrobisturí y los diferentes dispositivos de tecnología láser. La información disponible sobre estos instrumentos sugiere que la respuesta del tejido intervenido está influenciada por los principios físicos y biológicos de corte del instrumento.

**Objetivo.:**

Comparar los cambios histológicos en muestras de encía asociados al corte quirúrgico realizado con láser de diodo de 940 nm, láser de erbio, cromo: itrio-escandio-galio-granate (Er,Cr:YSGG) (2780nm) y electrobisturí mediante una presentación de serie de casos.

**Presentación de los casos.:**

Se presentan tres casos de pacientes sanos sometidos a cirugía estética. El examen clínico reveló la exposición de una banda gingival queratinizada mayor de 4 mm, tejido gingival de color y textura normales, de consistencia firme y sin sangrado al sondaje periodontal. Se indicó gingivectomía con los siguientes protocolos: láser de diodo de 940 nm a 1 W, en modo continuo; láser de Er,Cr:YSGG de 2780 nm a 2,5 W, 75 Hz, modo H, aire 20, agua 40, punta de oro MT4; y bisturí eléctrico en modo de corte, a nivel de potencia cuatro. Se tomaron muestras de tejido gingival y se almacenaron en formaldehído al 10 % para su análisis histológico.

**Conclusión.:**

Los tres instrumentos de corte generaron cambios histológicos producidos por el efecto térmico; los principales fueron coagulación del colágeno y carbonización. La profundización del daño térmico causada por el láser de Er,Cr:YSGG de 2780 nm fue mucho menor que la generada por el electrobisturí y por el láser de diodo de 940 nm.

Today, aesthetics is one of the most frequent reasons for patient’s dental visits ^1^. It is common to find cases of gummy smiles, where the patient manifests dissatisfaction due to the reduced size of the teeth and the amount of gingiva shown when smiling ^2^. If the primary cause of the gummy smile is periodontal, the conventional treatment seeks to increase the clinical crown of the teeth through a gingivectomy ^3^. This procedure is traditionally performed with a scalpel or electric scalpel. However, with the advent of new technologies in dentistry, laser technology could represent a surgical alternative with multiple benefits, and its application, for years, has been reported by different authors ^4^^-^^6^.

The 940 nm diode laser and the 2780 nm erbio, cromo: itrio-escandio-galio-granate (Er,Cr:YSGG) laser have entered the dental field with a wide range of applications in different areas ^7^^,^^8^. Its main benefits in soft tissue surgeries are reduction or absence of intraoperative bleeding, speeded healing, reduction of postoperative pain and inflammation, absence of risk of damaging the adjacent tooth or bone, excellent hemostasis, reduction of postoperative bacterial contamination, and therefore, reduced risk of infection, light contact of the fiber tip with the tissue, proprioceptive feedback and good patient acceptance, especially in those procedures requiring only local anesthesia ^9^^,^^10^.

A controlled clinical trial, carried out by Sobouti *et al.* in 30 patients who underwent gingivectomy to correct a gummy smile, showed that patients treated with the 940 nm diode laser had a significantly lower bleeding rate (0.36 out of 4) than that observed in the group operated with a conventional scalpel (1.15 out of 4). Likewise, it was observed that none of the diode laser participants showed post-surgical pain, nor did they consume analgesics, while in the control group, an average pain of 5.2 was obtained (out of a scale of 10), and 14 of the participants had to take pain medication ^11^.

This precedent shows the relevance of the instrument choice for soft tissue surgeries to provide comfort and results to the patient. Therefore, it is important to consider some aspects such as intraoperative and postoperative symptoms and time and mechanism of tissue healing, directly related to the effect exerted by the instrument to perform the cut.

From this point of view, an incision in the gingiva comprises numerous events depending on the instrument used, from damage to the cells and the extracellular matrix to the complete carbonization of the tissue, implying a greater difficulty in the wound repair and healing processes ^12^. Few studies report the histological effect on gingival tissue exerted by cutting instruments.

This article aims to compare the histological changes in gingiva associated with surgical cuts made with a 940 nm diode laser, a 2780 nm Er,Cr:YSGGE laser, and an electric scalpel through a series of cases.

## Case presentation

Three clinical cases are presented: Two female patients and one male, who attended the dental clinic of the *Corporación Universitaria Rafael Núñez* in Cartagena due to aesthetic discomfort. All patients reported: “I display a large amount of gum when smiling”.

**Figure 1:**
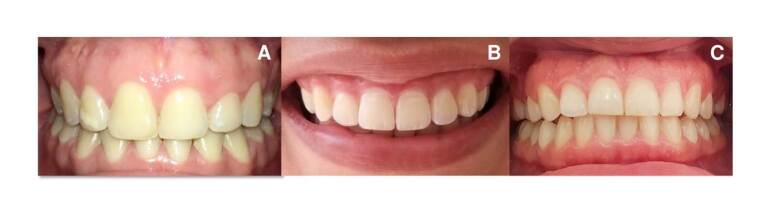
Initial photos. The clinical characteristics of the periodontium of each patient are observed.

During the anamnesis, patients did not report systemic pathologies or relevant personal or family medical history. When performing the stomatological examination, an exposure of keratinized gingiva greater than 4 mm was observed in the three cases. The tissue was healthy, of normal color and texture, with a firm consistency and no bleeding on periodontal probing (figure 1).

**Figure 2:**
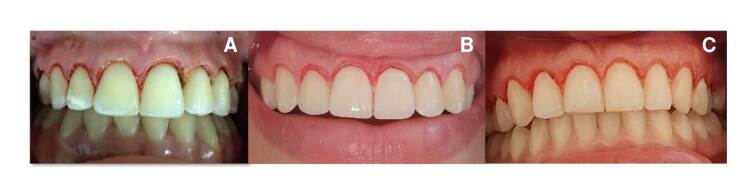
Images after the gingivectomy. **A.** Electric scalpel. **B.** 940 nm diode laser. **C.** 2780 nm Er,Cr:YSGG laser.

As a treatment plan, gingivectomy and gingivoplasty were indicated in the upper jaw. Before the surgical procedure, oral health education was carried out to reinforce hygiene habits. The procedures were performed as follows:

*Electrosurgical gingivectomy:* Two percent lidocaine plus 1:80,000 diluted epinephrine was used as local anesthesia. Probing was performed with a Michigan periodontal probe to determine the height and amount of gingival tissue to be cut. The cutting instrument used was an ART® Electrosurgery Unit in cut mode at an intensity level of four. The initial cut was made in the upper central incisors and used as guides to determine the gingival zenith. Then, we cut the lateral incisors, canines, and upper first premolars. At the end of the procedure, the patient was instructed to consume soft and cold foods for at least 24 hours. We prescribed drug therapy with 500 mg acetaminophen every six hours for three days. Clinical follow-up was carried out at 48 hours and 15 days after surgery (figure 2A).

*940 nm diode laser gingivectomy:* The area to be intervened was anesthetized with lidocaine spray. For the procedure, we used a BIOLASE® Epic X equipment, following a protocol of 1 W of power in a continuous mode with an E3-4 surgical tip (30 pm x 4 mm length), previously activated in articulating paper. At the end of the procedure, the patient was instructed to consume soft and cold food for at least 24 hours.

*2780 nm Er,Cr:YSGG laser gingivectomy:* Lidocaine spray was applied as topical anesthesia in the area to be operated. No infiltrative or regional anesthesia was used. We used a BIOLASE® Waterlase® equipment programmed at 2.5 W, 75Hz, H mode, air 20, water 40, and a gold sapphire tip MT4 (400 pm x 6 mm length). The procedure and tissue cutting were the same as those performed with the electric scalpel and diode laser (figure 2B and 2C).

In all cases, gingival tissue samples were taken and stored in 10% formaldehyde. Samples were sent to the histology laboratory for their examination using hematoxylin-eosin staining.

After the surgical procedures, follow-up was performed at eight days, observing satisfactory tissue response and absence of pain or sensitivity.

### Histological report

The histological findings associated with the instrument’s effects were evaluated in the three histological sections. We measured the estimated depth of thermal damage, taking as a reference the basal membrane as the upper limit and the deepest point with collagen degeneration. Measures were made with the digital image processing software Imagen G®, version 1.50i.

### Electric scalpel

**Figure 3:**
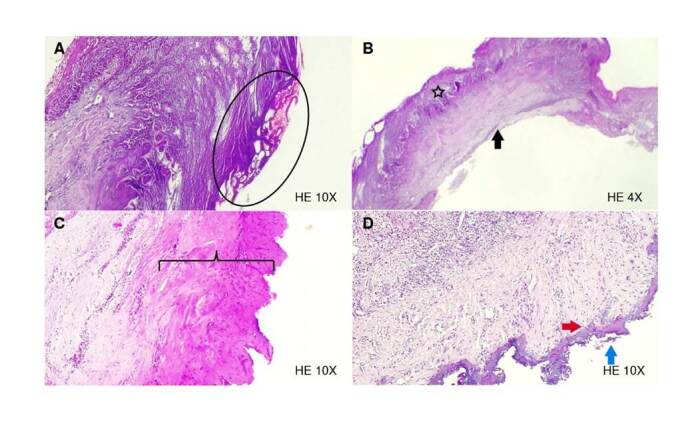
Histological sections. **A)** Electric scalpel: ovals indicate extensive burn changes. **B)** Electric scalpel: intercellular edema (star), and the deep resection margin shows a clear border (black arrow) with no evidence of carbonization. **C)** 940 nm diode laser: basophilic changes due to collagen degeneration (square brackets). **D)** 2780 nm Er,Cr:YSGG laser: fine homogeneous basophilic band (red arrowhead) and signs of carbonization with sloughing (blue arrow) of the tissue.

*Slide A (hematoxylin-eosin 10X):* The section shows a mucous membrane where the squamous epithelium predominates. Although it can be recognized, it has undergone extensive changes due to burns and heat (enclosed in the oval), leaving a large part of carbonized tissue (figure 3A). The estimated depth of thermal damage was 200 pm.

*Slide B (hematoxylin-eosin 4X):* This section shows gingiva with preserved mucosal epithelium, with some spongiotic changes - intercellular edema (star). The resection margin exposes a clear border (black arrow) with no evidence of carbonization or collagen degeneration (figure 3B).

*940 nm diode laser (hematoxylin-eosin 4X):* This section shows a gingiva fragment with preserved mucosal epithelium. Basophilic changes are evident due to collagen degeneration (in brackets), covering almost half of the tissue thickness in some areas. The estimated depth of thermal damage was 187 pm. The resection margin shows irregularity with a faint carbonization line (figure 3C).

*2780 nm Er, Cr: YSGG laser (H-E 10x):* The section reveals a deep resection margin with intense collagen degeneration extending only to the irregular connective tissue, evidenced by a thin homogeneous basophilic band (red arrowhead). The estimated depth of thermal damage was 27,2 pm. Fibroblasts preserved their morphology until the basophilic area. In epithelial tissue signs of carbonization are observed with tissue sloughing (blue arrow) and peripheral irregularity (figure 3D). Irregular dense connective tissue has abnormally dilated blood vessels. Regular dense connective tissue maintains its vascular network, fibroblasts have a fusiform appearance, and no degenerative changes are observed.

### Ethical considerations

The present case follows Resolution 008430 of 1993, which establishes scientific, technical, and administrative standards for health research. The three patients were informed about the procedure to be conducted, the risks and benefits derived from it, and voluntarily signed a consent form for the performance of the surgical procedure and the donation of their samples for histological analysis.

## Discussion

In dentistry, surgical cutting instruments have evolved. Currently, there are conventional scalpels, electric scalpels, and lasers, with varied applications in different dental specialties. All instruments incising the tissue generate lesions characteristic of its cutting mechanism and physical and biological properties ^13^. The cases presented in this paper confirm that the histological findings and the clinical characteristics of the wound vary according to the cutting equipment used. Although all instruments generate tissue lesions associated with thermal damage, when comparing them, we observed that some produce more damage than others.

Of the exposed cases, the 2780 nm Er,Cr:YSGG laser was the instrument that generated the least thermal damage. Cercadillo *et al*. confirmed it: The samples with less thermal effect were irradiated with Er,Cr:YSGG laser at 2780 nm and water/air spray, followed by the CO_2_ laser and 830 nm diode laser, concluding that the emission parameters of each system can impact the thermal damage inflicted on soft tissues. However, the wavelength of each laser influences tissue absorption rate and its reaction ^14^. This instrument superficial thermal effect in comparison with the electric scalpel and diode laser, lies in the spray of water attached to the cutting piece. According to its mechanism, the water is energized with the laser light, favors the cut, and serves as a cooling medium, contributing to tissue protection ^15^.

On the other hand, one of the samples taken with an electric scalpel revealed areas with tissue damage due to carbonization, while the second sample exposed cell preservation, and the thermal effect did not carbonize the tissue. This behavior suggests other variables influencing the tissue during cutting, such as the operator’s handling and the patient’s periodontal phenotype. A thick periodontal phenotype may imply more than one cut in the gingival margin to remove it completely. Therefore, the tissue will suffer more damage.

Regarding the effects of the electric scalpel, Ismail *et al*. indicated this incision produces thermal damage that includes carbonization, vacuolar degeneration, and nuclei elongation of the epithelial cells at the margins of the excision. Relevant findings with potential use in histopathological studies ^16^.

The 940 nm diode laser is a device with many indications in the dental field. Although it generated a thermal effect, we observed mucosa preservation and a poor carbonization area. Jin *et al.* compared wound healing after incisions with a conventional scalpel, a diode laser, and an Er,Cr:YSGG laser in the oral mucosa of guinea pigs and showed that the diode laser is a suitable device to use, but it produced more tissue damage than the scalpel or the Er,Cr:YSGG laser ^17^^,^^18^.

The clinical importance of these findings lies in the link between the histological condition and the postoperative period during the wound repair process ^19^. The epithelium healing route comprises a series of events in which the cellular and molecular dynamics of the injured tissue participate, seeking to restore integrity through epithelial migration that can be affected by different factors ^20^. During epithelium regeneration, the carbonization of the gingiva by the cutting instrument necessarily implies the necrosis of the burned area. The necrotic tissue acts as a barrier that interferes with the reparative action of the cells. Inflammation increases as leukocytes remove tissue debris through phagocytosis and lysis. In addition, necrotic tissue constitutes a niche for the proliferation of bacteria, increasing the risk of infection ^21^.

Some reported biological effects of the laser could improve or counteract the thermal damage generated by the irradiation of the gingival tissue during the cut. The laser light absorbed by the cells enhances ATP production, which induces cell migration and proliferation. On the other hand, the photobiomodulation effect of the laser promotes angiogenesis and local lymphatic drainage, leading to a decrease in inflammation and edema ^22^. These effects could be related to pain reduction, inflammation, and postoperative edema in patients treated with diode laser. Likewise, it presents an advantage over other instruments, such as the electric and the conventional scalpel.

Kaur *et al*. compared the efficacy and healing of soft tissue wounds using a diode laser (810 nm) versus the conventional scalpel approach in the second stage of implant surgery. They reported that laser use can minimize surgical trauma, reduce the amount of required anesthesia, improve visibility during surgery due to the absence of bleeding, and eliminate postoperative discomfort ^23^.

## Conclusions

Under the parameters used in this study, the three cutting instruments generated histological changes produced by the thermal effect. The main ones were collagen coagulation and carbonization. According to our measurements, the Er,Cr:YSGG laser was the instrument that generated the least thermal damage to the tissue. Therefore, more studies are needed to confirm these results and allow a broad comparison between the three instruments.

Despite these findings, we found cell preservation indicators, implying other factors not related to the instrument that probably influence the generated effect on the tissue. These factors could be the periodontal characteristics of each patient and the operator’s skill during the procedure.
